# EBI2 is a negative modulator of brown adipose tissue energy expenditure in mice and human brown adipocytes

**DOI:** 10.1038/s42003-022-03201-6

**Published:** 2022-03-29

**Authors:** Francesca Copperi, Inna Schleis, Martin Roumain, Giulio G. Muccioli, Stefano Casola, Martin Klingenspor, Alexander Pfeifer, Thorsten Gnad

**Affiliations:** 1 Institute of Pharmacology and Toxicology, University Hospital Bonn, University of Bonn, Bonn, 53127 Germany; 2 Research Training Group 1873, University of Bonn, Bonn, 53127 Germany; 3 Bioanalysis and Pharmacology of Bioactive Lipids Research Group, Louvain Drug Research Institute, UCLouvain, Université catholique de Louvain, 1200 Bruxelles, Belgium; 4 The FIRC Institute of Molecular Oncology (IFOM), Milan, 20139 Italy; 5 Molecular Nutritional Medicine, TUM School of Life Sciences, Technical University of Munich, 85354 Freising, Germany; 6 EKFZ—Else Kröner-Fresenius Center for Nutritional Medicine, Technical University of Munich, 85354 Freising, Germany; 7 ZIEL—Institute for Food & Health, Technical University of Munich, 85354 Freising, Germany

**Keywords:** Fat metabolism, Extracellular signalling molecules

## Abstract

Pharmacological activation of brown adipose tissue (BAT) is an attractive approach for increasing energy expenditure to counteract obesity. Given the side-effects of known activators of BAT, we studied inhibitors of BAT as a novel, alternative concept to regulate energy expenditure. We focused on G-protein-coupled receptors that are one of the major targets of clinically used drugs. Here, we identify GPR183, also known as EBI2, as the most highly expressed inhibitory G-protein-coupled receptor in BAT among the receptors examined. Activation of EBI2 using its endogenous ligand 7α,25-dihydroxycholesterol significantly decreases BAT-mediated energy expenditure in mice. In contrast, mice deficient for EBI2 show increased energy dissipation in response to cold. Interestingly, only thermogenic adipose tissue depots — BAT and subcutaneous white adipose tissue —respond to 7α,25-dihydroxycholesterol treatment/EBI2 activation but not gonadal white fat, which has the lowest thermogenic capacity. EBI2 activation in brown adipocytes significantly reduces norepinephrine-induced cAMP production, whereas pharmacological inhibition or genetic ablation of EBI2 results in an increased response. Importantly, EBI2 significantly inhibits norepinephrine-induced activation of human brown adipocytes. Our data identify the 7α,25-dihydroxycholesterol/EBI2 signaling pathway as a so far unknown BAT inhibitor. Understanding the inhibitory regulation of BAT might lead to novel pharmacological approaches to increase the activity of thermogenic adipose tissue and whole body energy expenditure in humans.

## Abstract (web summary)

Francesca Copperi et al. evaluate the role of the G_i_-protein coupled receptor, EBI2, on regulation of thermogenic activity in murine and human adipocytes. They report that loss of Ebi2 in mice increases brown adipocyte energy expenditure in response to cold exposure, providing insight into ways to potentially modulate energy expenditure in humans.

## Introduction

In mammals, two types of adipose tissue can be distinguished, the white adipose tissue (WAT) and the brown adipose tissue (BAT), which have different morphology, distribution, gene expression, and function. Brown adipocytes are characterized by multilocular morphology with a large number of small lipid droplets, high mitochondrial content and expression of the uncoupling protein 1 (UCP1). The main function of BAT is to dissipate the stored energy in form of heat in a process called non-shivering thermogenesis, which by activation of UCP1 uncouples ATP production leading to energy dissipation. WAT consists mostly of two major depots: subcutaneous (WATi) and gonadal (WATg). WATi—and to a much lesser extent also WATg—can adopt a brown-like phenotype/color in a process called browning or beiging^1–3^ in response to cold, to pharmacological stimulation (e.g. β-adrenergic or adenosine receptor stimulation)^4^, and several other stimuli. Oxysterols were long considered only as intermediates of cholesterol metabolism in the production of bile acids. Although the role of cholesterol^5,6^ and bile acids^7^ in brown/beige adipose tissue has been well described, not much is known about oxysterols in brown or beige fat.

Oxysterols signal via a broad spectrum of receptors including liver X receptors, insulin-induced gene proteins, retinoic acid receptor-related orphan receptors α and γ, as well as Epstein-Barr virus-induced gene 2 (EBI2)^8^. The latter is also known as GPR183, and belongs to the family of G-protein-coupled receptors (GPCRs), which are main regulators of brown fat physiology^4,9^. EBI2/GPR183 was first identified as a highly upregulated gene in B-cells infected with Epstein-Barr virus^10^. The endogenous ligand of EBI2 is the oxysterol 7α,25-dihydroxycholesterol (7α,25-OHC)^11,12^. EBI2 is involved in several immunological diseases like multiple sclerosis and inflammatory bowel disease^13,14^, but nothing was known about the role of EBI2 and its ligand 7α,25-OHC in BAT.

In this study, we identified the inhibitory, G_i_-protein-coupled EBI2 receptor as regulator of brown adipocyte activity. Our analysis revealed that EBI2 activation through its endogenous ligand 7α,25-OHC can potently decrease energy expenditure (EE), whereas the opposite effect can be obtained by loss of EBI2 or pharmacological blockade of the receptor. Overall, we demonstrated that EBI2 is a key regulator of whole-body metabolism in response to cold-mediated brown fat activation.

## Results

### EBI2 decreases brown adipocytes activation

**Fig. 1:**
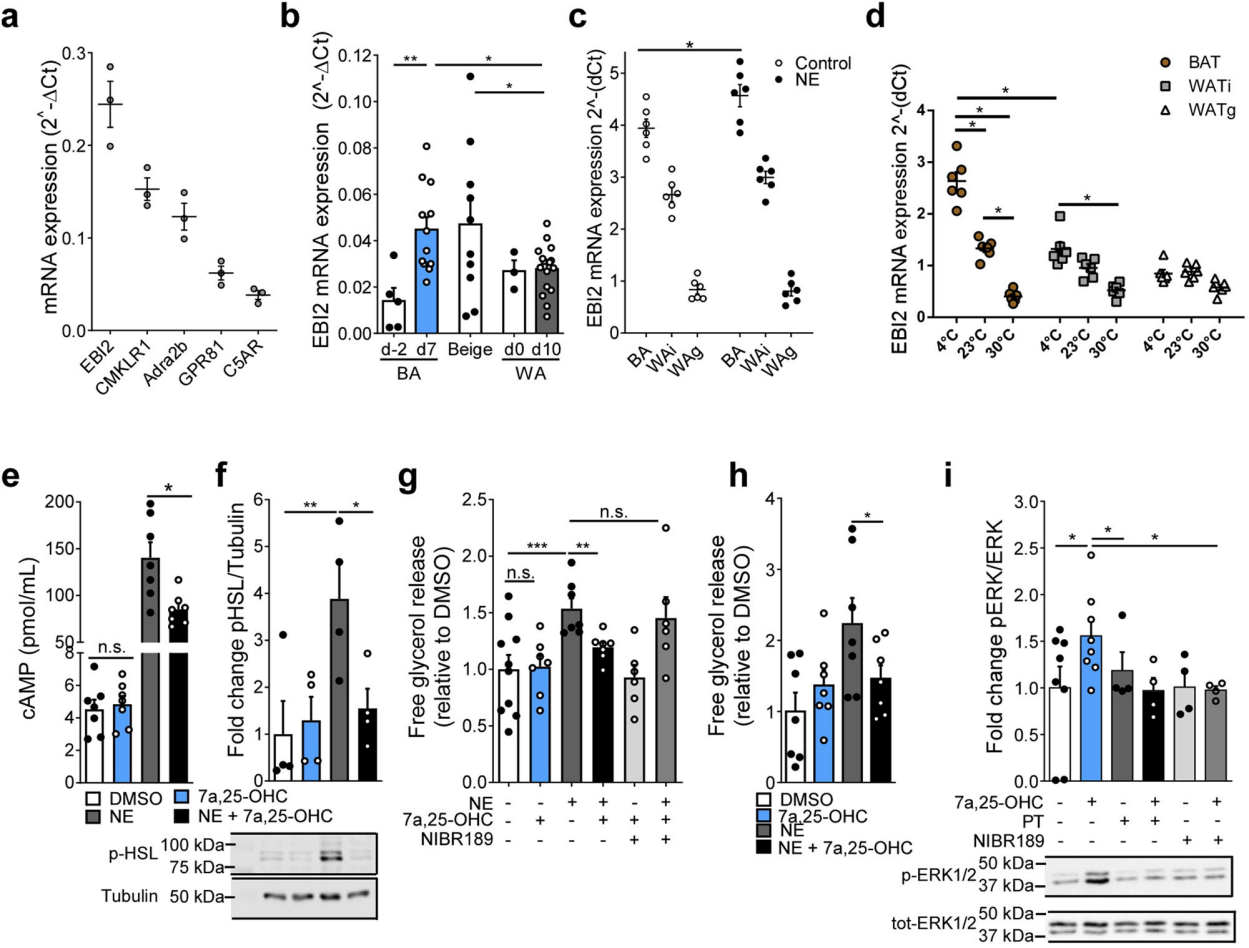
The effect of EBI2 stimulation on brown adipocytes and adipose tissues. **a** *Ebi2* and other highly expressed G_i_-coupled GPCRs mRNA expression in murine brown adipocytes (*n* = 3 independent cultures). **b** *Ebi2* mRNA levels in pre and mature brown (BA), beige and white (WA) adipocytes (*n* = 5 independent experiments). **c** *Ebi2* mRNA levels in BA, WAi, and WAg upon NE (1 µM) treatment (*n* = 6 independent experiments). **d** *Ebi2* mRNA levels in BAT, WATi and WATg following exposure to 4 °C, 23 °C and 30 °C (*n* = 6 mice per condition). **e** Intracellular cAMP levels in brown adipocytes treated with NE (1 µM) and 7α,25-OHC (0.1 µM) (*n* = 7 independent samples). **f** p-HSL representative immunoblot and relative quantification in brown adipocytes treated with NE (1 µM) and 7α,25-OHC (1 µM) (*n* = 4 independent experiments). **g** Relative lipolysis of brown adipocytes treated with NE (1 µM), 7α,25-OHC (1 µM) and NIBR189 (10 µM) (*n* = 6 independent samples). **h** Relative lipolysis of hMADs treated with NE (1 µM) and 7α,25-OHC (1 µM) (*n* = 7 independent experiments). **i** p-ERK/ERK representative immunoblot and quantification from brown adipocytes treated with 7a,25-OHC (1 µM), pertussis toxin (PT, 200 nM) and NIBR189 (10 µM) (*n* = 4 independent experiments) Mean ± s.e.m., one-way ANOVA with Tukey post-hoc test and Student’s *t* test, *p < 0.05, **p < 0.01, ***p < 0.001.

To unravel so far unknown GPCRs involved in the function of thermogenic fat, we screened for G_i_-coupled GPCRs highly expressed in BAT. In this study, we focus on the oxysterol receptor EBI2, because we found this receptor to be the most abundantly expressed receptor of the G_i_-coupled GPCRs analyzed in murine BAT (Fig. 1a and Supplementary Table 1). Further expression analysis revealed that *EBI2* mRNA is expressed in all adipose tissues, but abundance was highest in BAT and inguinal WAT (Supplementary Fig. 1a). Consistently, murine brown adipocytes expressed significantly higher levels of *EBI2* mRNA compared to white adipocytes (Supplementary Fig. 1b). *EBI2* mRNA expression was significantly higher in differentiated murine brown adipocytes compared to undifferentiated preadipocytes (Fig. 1b). Moreover, beige adipocytes expressed significantly more *EBI2* compared to white adipocytes (Fig. 1b). Thus, *EBI2* is more highly expressed in brown and beige then in white adipocytes suggesting a role of *EBI2* in thermogenic adipocytes. We next measured *EBI2* mRNA expression in adipocytes isolated from different fat depots as well as following norepinephrine (NE) stimulation to mimic cold exposure, which is the physiological stimulus for the activation of brown adipocytes and the development of a thermogenic program in white fat cells (“browning”) (Fig. 1c). NE treatment induced a significant increase of *EBI2* in brown fat cells, as well as an increased expression in adipocytes from WATi (Fig. 1c). No increase was observed in adipocytes from WATg (Fig. 1c). Cold exposure of mice induced a significant increase in *EBI2* in BAT, whereas thermoneutrality (30 °C) significantly decreased *EBI2* expression in BAT and WATi (Fig. 1d) suggesting the importance of EBI2 in regulating thermogenesis.

EBI2 was previously reported to signal via G_i_ and to decrease cAMP levels in CHO cells upon stimulation with 7α,25-OHC^12^ but nothing was known about its role in adipocytes. cAMP is a central second messenger in brown adipocytes that regulates lipolysis and EE^4,9^. Therefore, we analyzed EBI2 downstream signaling in brown adipocytes. Treatment with 7α,25-OHC induced a significant, dose-dependent decrease in intracellular cAMP of NE-stimulated murine brown adipocytes by 38% (Fig. 1e and Supplementary Fig. 1c). A similar inhibitory effect of 7α,25-OHC was observed in brown fat cells treated with the specific β_3_-adrenergic agonist CL-316,243 (Supplementary Fig. 1d). 7α,25-OHC also reduced cAMP levels in murine white fat cells, albeit not significantly and to a lesser extent than in brown adipocytes (Supplementary Fig. 1e). The cAMP/PKA signaling pathway regulates HSL phosphorylation, which in turn induces lipolysis—a hallmark of brown adipocyte activation. 7α,25-OHC treatment significantly reduced NE-stimulated HSL phosphorylation to levels similar to untreated brown adipocytes (Fig. 1f). To study the functional relevance of 7α,25-OHC/EBI2-mediated cAMP regulation, we analyzed lipolysis. 7α,25-OHC significantly reduced NE-mediated lipolysis in brown adipocytes (Fig. 1g) with an IC50 of only ~1.5 nM (Supplementary Fig. 1f). A similar significant effect was observed in brown adipocytes treated with 7α,25-OHC and CL-316,243 (Supplementary Fig. 1g). To analyze whether the effect of 7α,25-OHC signaling in brown adipocytes is mediated exclusively by EBI2, we used the highly specific EBI2 antagonist NIBR189^15^. Noteworthy, the effect of 7α,25-OHC on brown adipocyte lipolysis was completely blunted by NIBR189 (Fig. 1g and Supplementary Fig. 1g). Stimulation of EBI2 with 7α,25-OHC did not significantly alter NE-induced lipolysis in white fat cells (Supplementary Fig. 1h). Importantly, EBI2 activation decreased NE-induced (Fig. 1h) as well as CL-316,243-induced (Supplementary Fig. 1i) lipolysis also in the human brown/beige adipocyte cell line (hMADs), suggesting that 7α,25-OHC/EBI2 signaling regulates β_3_-adrenergic receptor-mediated activation of murine and human brown fat cells.

To further scrutinize EBI2 signaling, we first focused on ERK1/2, which has been shown to be activated by different G-protein α and βγ subunits^16^. EBI2 activation significantly induced ERK phosphorylation in murine brown adipocytes (Fig. 1i). To study whether EBI2 is coupled to G_i_ protein also in brown adipocytes, we used the G_i_-specific inhibitor pertussis toxin. Notably, pre-treatment of brown adipocytes with pertussis toxin abolished 7α,25-OHC-induced ERK1/2 phosphorylation demonstrating the coupling of EBI2 to G_i_ also in brown fat cells (Fig. 1i). Again, pharmacological blockade of EBI2 completely blunted 7α,25-OHC-induced ERK1/2 phosphorylation indicating that this effect is mediated by EBI2 signaling (Fig. 1i).

Altogether, these data show that 7α,25-OHC/EBI2 signaling decreases activation of brown adipocytes.

### 7α,25-OHC is an autocrine inhibitor of brown adipocyte activity

**Fig. 2:**
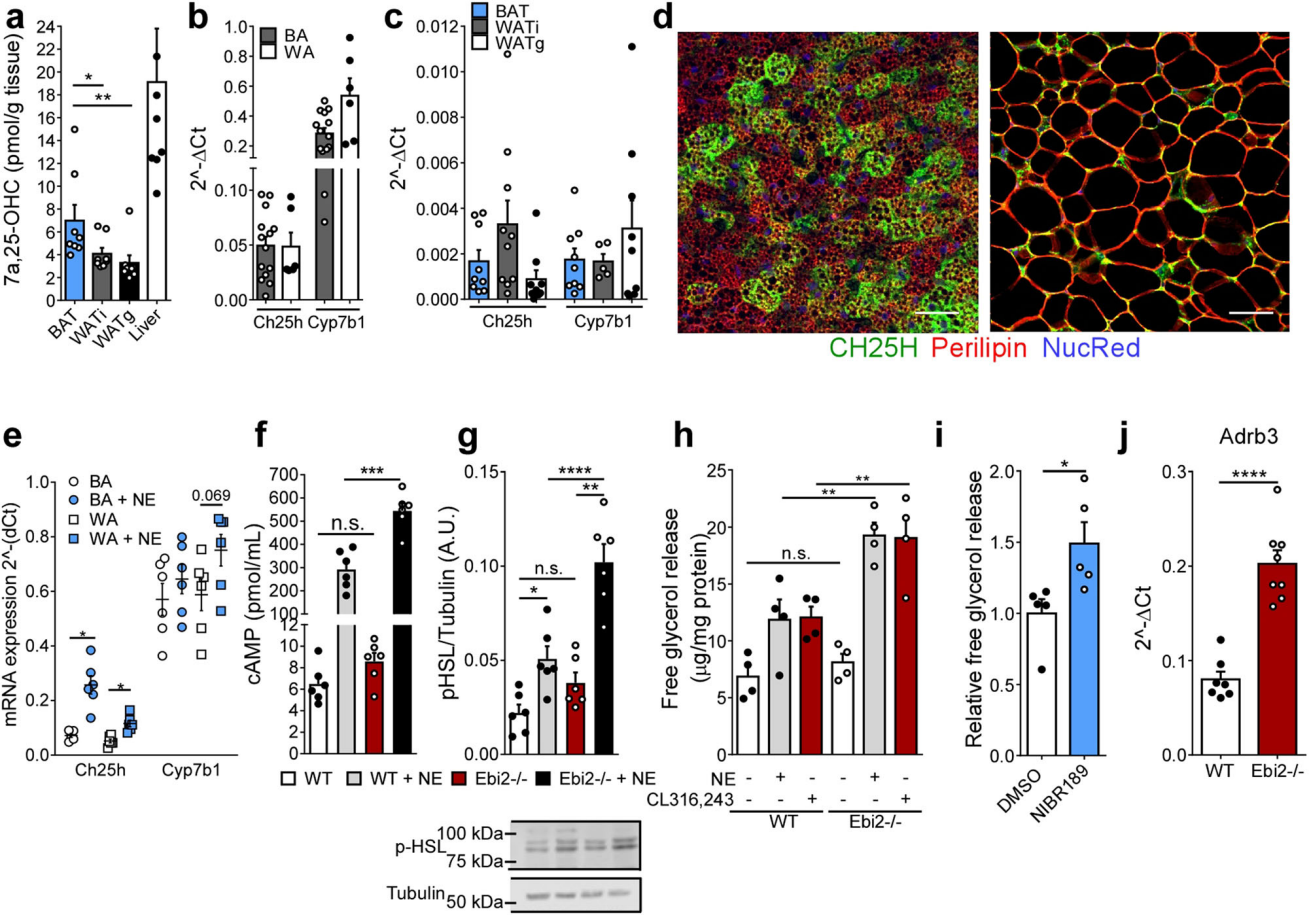
The source of 7a,25-OHC and its autocrine effects on brown adipocytes. **a** 7α,25-OHC levels in BAT, WATi, WATg, and liver (*n* = 8). **b** *Ch25h* and *Cyp7b1* mRNA levels in brown and white adipocytes (*n* = 5–14) **c** *Ch25h* and *Cyp7b1* mRNA levels in ATs (*n* = 10). **d** CH25H (green) and perilipin (red) stainings in BAT (left) and WATi (right) sections (Scale bar 50 µM). **e** *Ch25h* and *Cyp7b1* mRNA expression in brown and white adipocytes upon NE (1 µM) treatment (*n* = 5). **f** Intracellular cAMP levels in WT and EBI2−/− brown adipocytes treated with NE (1 µM) (*n* = 6). **g** p-HSL representative immunoblot and relative quantification in WT and EBI2−/− brown adipocytes treated with NE (1 µM) (*n* = 6). **h** Lipolysis of WT and EBI2−/− brown adipocytes treated with NE (1 µM) or CL316,413 (10 µM) (*n* = 4). **i** Lipolysis of WT brown adipocytes treated with NE (1 µM) and NIBR189 (10 µM) (*n* = 5). **j** *Adrb3* mRNA levels in WT and EBI2−/− brown adipocytes (*n* = 7–8). Mean ± s.e.m., one-way ANOVA with Tukey post-hoc test and Student’s *t* test, *p < 0.05, **p < 0.01, ***p < 0.001, ****p < 0.0001.

To study the physiological role of EBI2 in adipose tissue, we first measured the abundance of its endogenous ligand 7α,25-OHC in BAT and WAT with LC-MS. Noteworthy, 7α,25-OHC levels were higher in BAT and WATi compared to WATg (Fig. 2a). 7α,25-OHC is metabolized from cholesterol by two enzymes: 1) cholesterol-25-hyroxylase (CH25H) and 2) cholesterol-7-alpha-hydroxylase (CYP7B1). Interestingly, we found mRNA expression of both enzymes in brown and white adipocytes (Fig. 2b), as well as in all three main adipose tissue depots (Fig. 2c). Histological analysis revealed co-localization of CH25H and lipid droplet-marking perilipin in BAT (Fig. 2d, left) and WATi (Fig. 2d, right). Noteworthy, *CH25H* was significantly upregulated in both brown and white fat cells after sympathetic stimulation (Fig. 2e). *CYP1B1* was also upregulated in both types of adipocytes, albeit not significantly (Fig. 2e). These data show that the enzymatic machinery regulating 7α,25-OHC abundance is upregulated upon sympathetic stimulation/adipocyte activation. Moreover, the endogenous EBI2 ligand 7α,25-OHC is present in BAT and might signal to brown adipocytes in an auto-/paracrine manner.

### Increased NE signaling in EBI2-deficient brown adipocytes

To further study the role of EBI2 in BAT, we isolated brown adipocytes from mice deficient for EBI2 (EBI2−/−). Notably, NE-stimulated intracellular cAMP levels of EBI2−/− brown adipocytes were significantly increased by 86% compared to NE-treated wild-type (WT) brown adipocytes (Fig. 2f). Consequently, EBI2−/− brown fat cells showed significantly higher NE-induced HSL phosphorylation (Fig. 2g). Furthermore, lipolysis was significantly increased by 62% in EBI2−/− compared to WT brown adipocytes after NE treatment, and specific activation of the β_3_-adrenergic receptor using CL-316,243 significantly elevated glycerol release by 58% compared to WT (Fig. 2h). A higher response to NE treatment was also observed after pharmacological blockade of EBI2 (Fig. 2i). The increased activity of EBI2−/− brown adipocytes compared to WT following NE stimulation supports the hypothesis of an autocrine activation of EBI2 by brown adipocytes via 7α,25-OHC production. The observed differences between EBI2−/− and WT brown adipocytes were not due to changes in cell differentiation, as demonstrated by comparable levels of thermogenic (Supplementary Fig. 2a, b) and adipogenic (Supplementary Fig. 2c) markers. Interestingly, EBI2−/− brown adipocytes showed also significant higher expression of β_3_-adrenergic receptor mRNA (Fig. 2j). This might contribute to the increased effects of NE observed in EBI2−/− brown adipocytes (Fig. 2f–h) and after pharmacological blockade of 7α,25-OHC/EBI2 signaling (Fig. 2i).

### EBI2 signaling regulates ROS production

**Fig. 3:**
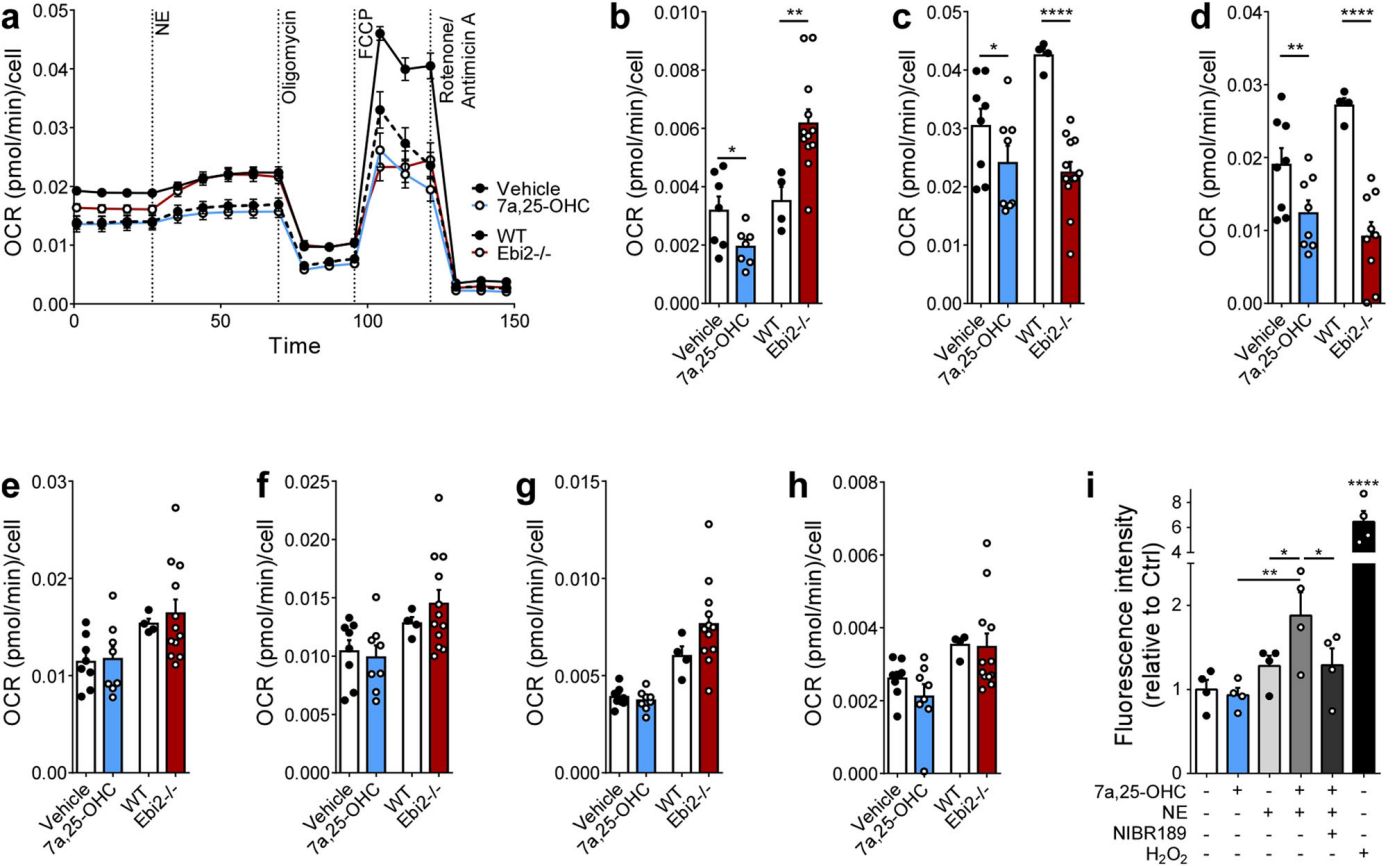
The effect of EBI2 signaling on brown adipocytes respirometry and ROS generation. **a** In vitro respirometry of brown adipocytes pre-treated with 7α,25-OHC (1 µM), and EBI2−/− brown adipocytes (*n* = 6 independent samples). **b**–**h** NE-induced O_2_ consumption (**b**), maximal respiration (**c**), spare respiratory capacity (**d**), basal respiration (**e**), ATP production (**f**), proton leak (**g**) and non-mitochondrial respiration (**h**) of brown adipocytes pre-treated with 7α,25-OHC (1 µM), and EBI2−/− brown adipocytes (*n* = 6 independent experiments). **i** ROS production in brown adipocytes after 1 h treatment with NE (1 µM), 7α,25-OHC (1 µM), NIBR189 (10 µM) or 3% H_2_O_2_ (*n* = 4 independent samples). Mean ± s.e.m., one-way ANOVA with Tukey post-hoc test and Student’s *t* test, *p < 0.05, **p < 0.01, ****p < 0.0001.

Oxygen consumption is a key measure of brown adipocyte activation. Hence, we studied mitochondrial respiration in brown adipocytes after 7α,25-OHC treatment and in EBI2−/− brown adipocytes. Noteworthy, NE-induced oxygen consumption was decreased by 40% and 12% in murine (Fig. 3a, b) and human (Supplementary Fig. 2d, e) brown fat cells, respectively, after 7α,25-OHC treatment. Conversely, loss of EBI2 resulted in a 75% higher mitochondrial oxygen consumption (Fig. 3a, b) when compared to WT murine brown fat cells. Moreover, 7α,25-OHC treated WT brown adipocytes, as well as EBI2−/− brown adipocytes, exhibited significantly reduced maximal respiration (i.e. oxygen consumption upon treatment with the protonophore FCCP) (Fig. 3c) as well as spare respiratory capacity (Fig. 3d) (calculated as difference between maximal and basal respiration), while no differences were observed in the basal respiration, ATP production, proton leak and non-mitochondrial respiration (Fig. 3e–h). These results show that EBI2 activation reduces NE-mediated energy dissipation of brown adipocytes, whereas EBI2 loss increases NE-induced oxygen consumption.

Previous publications have reported that treatment of brown adipocytes with NE acutely increases ROS production, and that ROS production is responsible for a decrease in maximal respiration^17,18^ and spare respiratory capacity in endothelial cells^19^. To investigate possible effects on ROS production, murine brown adipocytes were treated with 7α,25-OHC, NE and NIBR189, and the kinetic of ROS production was measured. The NE treatment increased ROS production, albeit not significantly, reaching a plateau after 60 min of treatment (Fig. 3i and Supplementary Fig. 2f). 7α,25-OHC alone had no effect on ROS production, however, ROS levels were significantly increased in the presence of NE (Fig. 3i and Supplementary Fig. 2f), with a peak at 60 min (Fig. 3i and Supplementary Fig. 2f). The 7α,25-OHC effect was blunted by NIBR189, thus indicating that the effect was mediated by EBI2 (Fig. 3i and Supplementary Fig. 2f).

These results show that 7α,25-OHC decreases the NE-induced oxygen consumption/maximal respiration and enhances NE-induced ROS production in murine brown adipocytes.

### Acute activation of EBI2 with 7α,25-OHC reduces energy expenditure

**Fig. 4:**
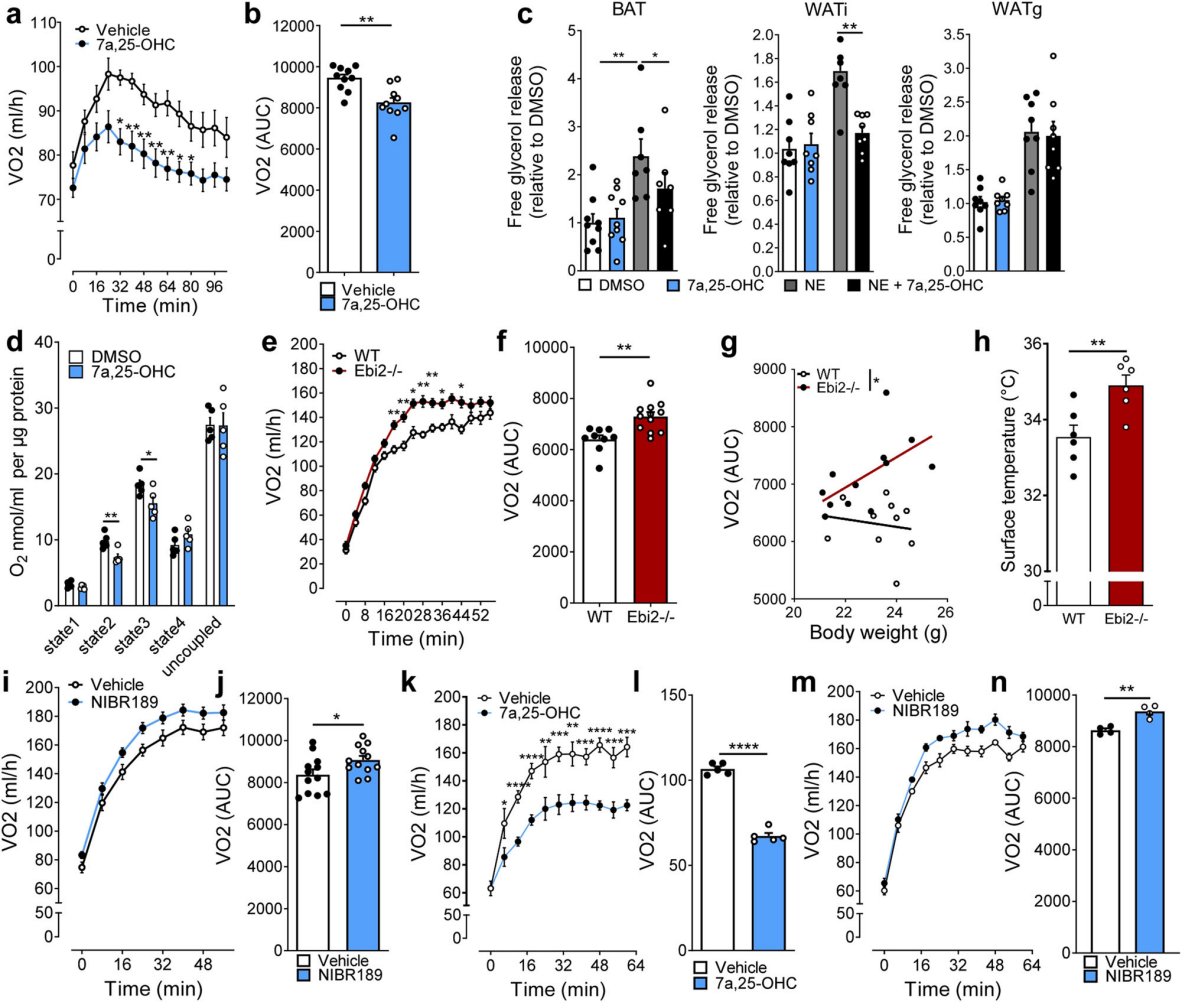
EBI2-mediated regulation of energy expenditure in vivo. **a**, **b** Energy expenditure (VO2) following injection of 7α,25-OHC (5 mg/kg i.p.) or vehicle (*n* = 10 mice per condition). **c** Relative lipolysis in ATs (pups BAT, left; adult WATi, middle; adult WATg, right) treated with NE (1 µM) and 7a,25-OHC (1 µM) (*n* = 8 independent explants per condition). **d** Ex vivo oxygen respiration of BAT treated with vehicle or 7α,25-OHC (1 µM) (*n* = 5 independent samples). **e**, **f** Energy expenditure (VO2) of WT and EBI2−/− mice during 1 h of cold exposure (4 °C) (*n* = 10 WT and *n* = 12 Ebi2−/−). **g** ANCOVA analysis of energy expenditure and body weight of WT and EBI2−/− mice during 1 h of cold exposure (*n* = 10 WT and *n* = 12 Ebi2−/−). **h** Infrared thermography of WT and EBI2−/− newborn mice at 23 °C (*n* = 6 pups per genotype). **i, j** Energy expenditure (VO2) of mice treated with vehicle or EBI2 antagonist NIBR189 during 1 h at 4 °C (*n* = 12 animals per condition). **k**, **l** Energy expenditure (VO2) over 1 h at 16 °C of mice injected with vehicle or 7α,25-OHC (*n* = 5 mice per condition). **m**, **n** Energy expenditure (VO2) over 1 h at 16 °C of mice injected with vehicle or NIBR189 (*n* = 4 per condition). Mean ± s.e.m., two-way ANOVA, ANCOVA linear regression and Student’s *t* test, *p < 0.05, **p < 0.01, ***p < 0.001, ****p < 0.0001.

To analyze the role of 7α,25-OHC/EBI2 signaling on whole-body EE, WT, and EBI2−/− mice were injected with 7α,25-OHC (5 mg/kg, i.p.) and oxygen consumption was measured. 7α,25-OHC-treated WT mice showed a significantly reduced (−13%) EE compared to control animals (Fig. 4a, b and Supplementary Fig. 3a, b) without affecting body weight (Supplementary Fig. 3c). Noteworthy, EBI2−/− mice showed no significant change in EE after receiving 7α,25-OHC indicating that effects of 7α,25-OHC on whole-body EE are mediated by EBI2 (Supplementary Fig. 3d–g). Moreover, treatment with 7α,25-OHC resulted in significantly decreased NE-stimulated lipolysis in BAT and WATi explants (Fig. 4c). However, 7α,25-OHC did not have a significant effect in WATg—the depot with the lowest browning capacity (Fig. 4c), suggesting EBI2 signaling to be specific for thermogenic adipose tissues. Consistently, 7α,25-OHC treatment decreased mitochondrial respiration of BAT (Fig. 4d).

Altogether, these data show that 7α,25-OHC/EBI2 signaling decreases whole-body EE by decreasing BAT activation.

### Loss of EBI2 enhances acute energy expenditure in response to cold

Acute cold stimulates the sympathetic nervous system leading to NE release and subsequent β-adrenergic receptor-mediated BAT activation. Given higher NE-induced lipolysis and oxygen consumption in EBI2−/− brown adipocytes, we studied the role of EBI2 in BAT-dependent EE in vivo. Prior to cold exposure, EBI2−/− and WT littermates housed at 23 °C showed no differences in EE (Supplementary Fig. 4a–d), body weight (Supplementary Fig. 4e), adipocyte morphology (Supplementary Fig. 4f), BAT UCP1 levels (Supplementary Fig. 4g), adipogenic and thermogenic gene expression in BAT and WATi (Supplementary Fig. 4h), serum glucose (Supplementary Fig. 4i) and serum insulin (Supplementary Fig. 4j). However, when BAT was challenged with acute cold exposure, EBI2−/− mice had a significantly (+20%) greater increase in whole-body EE compared to WT littermates (Fig. 4e, f and Supplementary Fig. 5a, b) independently of body weight (Fig. 4g). Levels of 7α,25-OHC (Supplementary Fig. 5c) and thermogenic marker expression (Supplementary Fig. 5d) were not affected after this short cold exposure. Newborn mice strongly rely on BAT to sustain body temperature. Interestingly, EBI2−/− newborns had a significantly higher surface temperature in the interscapular area compared to WT littermates (Fig. 4h). In line with the data from the EBI2−/− mice, a similar increase in EE was observed in cold-exposed WT mice after pharmacological blockade of EBI2 (Fig. 4i, j and Supplementary 5e, f). Given the role of EBI2 in the immune system, we analyzed cold-induced oxygen consumption in mice lacking EBI2 specifically in monocytes/macrophages (M-EBI2−/−): notably, no differences were detected in oxygen consumption between M-EBI2−/− compared to control mice (Supplementary Fig. 5g–j).

To check for beige fat contribution to EE (i.e. WATi after prolonged cold exposure), we housed EBI2−/− and WT mice for 1 week at 4 °C and measured oxygen consumption. No difference in EE was observed after prolonged cold exposure (Supplementary Fig. 6a–d) in EBI2−/− compared to WT mice, nor in body weight (Supplementary Fig. 6e) and adipose tissues weight (Supplementary Fig. 6f). In this context, it is of interest that expression of β_3_-adrenergic receptor mRNA was significantly lower in WATi of EBI2−/− mice compared to WT (Supplementary Fig. 6g).

Finally, we checked whether stimulation of EBI2 was able to counteract cold-induced EE/BAT activity. Treatment with 7α,25-OHC significantly decreased oxygen consumption compared to vehicle-treated mice (Fig. 4k, l). Consistently, EBI2 antagonism increased EE after cold exposure (Fig. 4m, n). Both 7α,25-OHC (Supplementary Fig. 6h, i) and NIBR189 (Supplementary Fig. 6j, k) treatments were not effective in modulating BAT-mediated thermogenesis in EBI2−/− mice, indicating their effect is indeed mediated by EBI2.

Together, these data indicate that EBI2 is a potent regulator of acute cold-induced EE, and the lack of EBI2 or its pharmacological inhibition is able to increase whole-body oxygen consumption.

## Discussion

In the last decade, *EBI2*, also known as *GPR183*, has been shown to be a major player in multiple immunopathological conditions^14^. However, nothing was known about the presence and function of *EBI2* in adipose tissues. In the present study, we show that *EBI2* is highly expressed in BAT and that it is a major regulator of BAT activity: stimulation of EBI2 with its endogenous ligand 7α,25-OHC reduced cold-induced EE. Vice versa, genetic deletion of *EBI2* as well as pharmacological blockade significantly increased whole-body EE in vivo.

In white fat, G_i_-coupled GPCRs like adenosine receptor A1 or GPR81 have been shown to decrease lipolysis^20,21^. However, not much is known about inhibitory, G_i_-coupled GPCRs in the regulation of BAT. Sphingosine 1 phosphate receptors, which signal via G_i_ proteins have been described to regulate BAT mass, however, no effect on BAT activation was reported^22^. Interestingly, the G_i_-coupled fatty acid receptor GPR43 has been shown to inhibit lipolysis in WA^23^, but activation of GPR43 increased oxygen consumption in BAT^24^. Thus, GPCRs that couple to G_i_ can have different effects in white versus brown adipocytes and one cannot directly infer from one type of fat to the other. A recent study^25^ found no effect on basal lipolysis of G_i_-coupled designer receptors exclusively activated by designer drugs in absence of G_s_-coupled receptor activation. Similarly, we detected no significant effect under basal conditions after activation of EBI2 indicating that the low basal levels of cAMP are insufficient to be significantly decreased by a single G_i_-coupled GPCR. On the other hand, brown adipocytes deficient for Gα_i2_ proteins exhibit enhanced oxygen consumption already under basal conditions^26^.

Our study clearly shows that EBI2 couples to G_i_ proteins in brown and white adipocytes similar to what was observed in other cell types^11,27^. Activation of EBI2 potently decreased NE- as well as β_3_-adrenoceptor specific induced lipolysis in brown adipocytes. Since NE is the major physiological activator of BAT and thermogenesis, these data indicate that EBI2 might constitute a biologically important inhibitor of BAT activation. In line with this notion, EBI2 activation dampened activation of BAT in acute cold exposure.

Interestingly, the levels of the endogenous EBI2 ligand 7α,25-OHC were significantly higher in BAT than in WAT. Moreover, *EBI2* mRNA expression was significantly lower in white than in brown fat cells. Accordingly, EBI2 activation did not significantly decrease NE-induced lipolysis and intracellular cAMP in white adipocytes. In contrast, 7α,25-OHC treatment decreased NE-induced lipolysis in BAT and WATi, the two thermogenically active adipose tissues, but not in WATg suggesting a specific role of EBI2 in modulating EE and thermogenesis. Not much is known about oxysterols or 7α,25-OHC in particular in thermogenic fat. Previous work showed the presence of 4β-hydroxyoxysterol and 27-hydroxycholesterol in WATi. Interestingly, the abundances of these two oxysterols are increased in diet-induced and genetic mouse models of obesity^28,29^, which might have inhibiting effects on the browning capacity of this white fat depot. Nothing has been described about the source of 7α,25-OHC, the endogenous EBI2 ligand, in thermogenic adipose tissues. We found significantly higher levels of 7α,25-OHC in BAT compared to WATi and WATg of mice housed at 23 °C. Interestingly, we found *CH25H*—a crucial rate limiting enzyme of 7α,25-OHC abundance—significantly upregulated in NE-stimulated brown fat cells.

Previous work indicated that ROS production in BAT is essential for mitochondrial activity^17^, but that an excessive increase in ROS leads to a decrease in mitochondrial respiration^30^. In line with these findings, we found NE-stimulated mitochondrial ROS production in brown adipocytes. Surprisingly, when brown adipocytes were treated with both NE and 7α,25-OHC, the increase in ROS levels was significantly higher than in the 7α,25-OHC or NE-treated cells. This may be caused by the corresponding decrease in lipolytic activity, along with a lower UCP1-dependent leak respiration rate induced by 7α,25-OHC. At low (or no) UCP1 activity, superoxide production is high, as shown in isolated BAT mitochondria^31^ and in BAT in vivo^30^. Our data support the model suggesting that inactive UCP1 promotes mitochondrial superoxide production to sensitize the responsivity to NE and support thermogenesis. Once activated, however, UCP1 mitigates superoxide production and thereby prevents excess ROS production at high rates of thermogenesis.

Consistently with the in vitro data presented here, the acute in vivo activation of EBI2 by 7α,25-OHC injection decreased the whole-body metabolism of mice, resulting in significant decreased oxygen consumption and EE in the first hour following the injection. Previous studies reported that the majority of oxysterols have a very short half-life compared to cholesterol in vivo, (i.e. <1 h)^32,33^, offering a possible explanation why 7α,25-OHC elicits an acute reduction of cold-induced BAT activity/EE.

In line with the effect of EBI2 stimulation on BAT activity, loss of EBI2 significantly increased acute cold-induced EE. Importantly, a similar increase in EE was obtained by injecting the EBI2 selective antagonist NIBR189. Thus, pharmacological inhibition of EBI2 in BAT might be a possible therapeutic approach to increase whole-body EE in humans. The short time window (<2 h) in which modulation of EBI2 activity was able to affect EE might be interesting from a therapeutic point of view for the treatment of obese human subjects. Recent studies showed that cryotherapy applied to obese patients for <2 h was able to significantly reduce BMI and body fat^34^. Further studies will address whether inhibition of EBI2 in obese human subjects might be beneficial for increasing the effects of cold therapy for the treatment of obesity.

In summary, our data demonstrate that EBI2 and its endogenous ligand 7α,25-OHC are potent inhibitors of BAT activity, whereas loss of EBI2 significantly increases EE in response to cold-induced BAT activation. This insight in thermogenic fat physiology and oxysterols biology paves the way to new pharmacological treatments to modulate EE.

## Methods

### Isolation and differentiation of primary adipocytes

Primary BAs and WAs were isolated from newborn (unknown sex) and 8–10 weeks old (male) mice respectively as described previously^35^. Briefly, excised interscapular BAT of newborn mice was digested with collagenase for 30 min at 37 °C and filtered. The mesenchymal stem cells fraction was re-suspended and seeded on a 6-well plate. The preadipocytes were then immortalized with SV40 large T antigen and unselected cells were cultured, expanded up to 4 passages and cryopreserved. For differentiation, immortalized cells were seeded on the appropriate plate (6 or 12-well) in growth medium (DMEM supplemented with 10% FBS and 0.1% Penicillin/Streptomycin). After 2 days, medium was changed to differentiation medium (growth medium, 20 nM insulin and 1 nM triiodothyronine). After 2 other days, differentiation was induced with induction medium (differentiation medium, 0.5 mM isobutylmethylxanthine (IBMX) and 1 μM dexamethasone). Following 2 days of induction, the medium was changed to differentiation medium and replenished every second day for other 5 days. All the treatments were performed 7 days after induction.

For WAs, the subcutaneous fat pads were excised and digested with collagenase for 30 min at 37 °C. The pellet was filtered and cells were seeded until 80% confluence or 7 days in growth medium, in 175 T flasks and cryopreserved. For differentiation, preadipocytes were seeded in growth medium and differentiation was induced 2 days after confluence with induction medium (DMEM supplemented with 5% FBS and 0.1% Penicillin/Streptomycin, 1 μM dexamethasone, 0.5 mM IBMX, 1 nM triiodothyronine, 1 mM D-biotin, 17 mM pantothenate, L-ascorbate (50 mg/ml), 1 μM rosiglitazone and 0.172 mM insulin). After 2 days the medium was changed to maintenance medium (DMEM supplemented with 5% FBS and 0.1% Penicillin/Streptomycin, 1 nM triiodothyronine, 1 mM D-biotin, 17 mM pantothenate, L-ascorbate (50 mg/ml) and 0.172 mM insulin) and replenished every second day for 10–13 days. Beige cells were obtained from WAs treated with Norepinephrine (1 µM) for 16 h.

### hMADs differentiation

Human multipotent adipose-derived stem cells were provided by the laboratory of C. Dani (University of Nice SophiaAntipolis)^36^ and differentiated as described previously^35^. Briefly, 160 000 cells were seeded on 12-well plates in growth medium (DMEM Low Glucose (Lonza), supplemented with 1 × glutamine (Lonza), 10 mM Hepes buffer (Lonza), penicillin-streptomycin 5000 IU/ml to 5000 UG/ml (Lonza) and 10% FBS (S.A Dutscher, Brumath, France)) containing 2.5 ng/ml FGF2 (Peprotech). After 48 h, medium was replaced with growth medium without FGF2. When confluence was reached, growth medium was replaced by hMADS induction medium (day 0) (growth medium supplemented with 5 μg/ml insulin, 10 μg/ml transferrin, 0.2 nM triiodothyronine, 1 μM rosiglitazone, 100 μM IBMX and 1 μM dexamethasone) for the next 72 h. Cells were then cultured in differentiation medium (induction medium without IBMX and dexamethasone) for 9–11 more days.

### RNA isolation and real-time RT-qPCR

Total RNA was isolated using Trizol method (Analytik Jena AG). cDNA was synthesized from 0.5-1 μg RNA using ProtoScript II First Strand cDNA Synthesis Kit (New England Biolabs). Real-time RT-PCR (qPCR) was performed with SYBR-Green PCR master mix (Applied Biosystems) using a HT7900 instrument (Applied Biosystems) or a QuantStudio 5 instrument (Applied Biosystems). Fold changes were calculated using relative quantification methods with mHprt (murine hypoxanthine guanine phosphoribosyl transferase) or mActb (murine actin beta) serving as an internal control.

### Protein isolation and western blot analysis

Protein lysates from cells and tissues were isolated using lysis buffer (50 mM Tris, pH 7.5, 150 mM sodium chloride, 1% NP-40, 0.5% sodium deoxycholate, 0.1% SDS, 0.1 mM EDTA and 0.1 mM EGTA) supplemented with complete protease inhibitor cocktail (Roche), 1 mM Na_3_VO_4_ and 10 mM NaF. Protein content was determined with the Bradford method. Proteins were separated using SDS-polyacrylamide gel electrophoresis and transferred onto a nitrocellulose membrane with a Trans-Blot Turbo Transfer System (Bio-Rad) and Towbin buffer (25 mM Tris base, 192 mM glycine, 20% v/v methanol, 0.1% SDS). Membrane was blocked for 1 h in 5% BSA or 5% non-fat milk, according to antibody manufacturer instruction, in Tris-Buffered Saline and 0.1% Tween 20 (TBST) and incubated overnight at 4 °C in different primary antibodies (p-HSL (1:1000, Cell Signaling), p-ERK1/2 and tot-ERK1/2 (1:1000, Cell Signaling), UCP1 (1:1000, Sigma-Aldrich), Tubulin (1:1000, Dianova), Calnexin (1:2000, Novus Biologicals). Incubation in secondary antibody (HRP-conjugated or DyLight 800-conjugated, Cell signaling) was performed the next day, for 1 h at room temperature in TBST. Proteins were visualized with enhanced chemiluminescence (ECL) reagent and quantified by densitometric analysis with Image J software or detected and quantified with the Odyssey Fc Imaging System.

### cAMP ELISA

Intracellular cAMP was determined by Direct-cAMP ELISA kit (Enzo Life Sciences) according to manufacturer instructions. BAs and WAs were treated for 30 min with 3-isobutyl-1-methylxanthine (100 μM), 15 min with 7α,25-OHC, and 15 min with norepinephrine (1 μM, Sigma Aldrich). All measurements were run in duplicate. Results were normalized to protein content measured with the Bradford method.

### Lipolysis assay

Differentiated adipocytes were washed twice with lipolysis medium (Gibco) supplemented with 2% w/v fatty acid–free BSA (Sigma-Aldrich) followed by incubation with lipolysis medium containing indicated substances at 37 °C and 5% CO_2_ for 2 h (for BA and WA) or 4 h (for hMADs). For ex vivo lipolysis assay, tissue explants were isolated from either newborn mice (BAT, unknown sex) or 8 weeks old mice (WATi and WATg, male) and incubated with lipolysis medium containing indicated substances at 37 °C and 5% CO_2_ for 2 h. Free glycerol release was measured with free glycerol reagent (Sigma-Aldrich) according to manufacturer instruction. Absorption was measured at 540 nM and 600 nM (EnSpire Multimode Plate Reader, Perkin Elmer) and glycerol release was calculated with glycerol standard (Sigma-Aldrich) normalized to protein content (for in vitro assay) or tissue weight (for ex vivo assay).

### In vitro respirometry

Immortalized primary BA was seeded in Seahorse 24-well plates (Agilent) 40 000 cells/well in 100 μl of BA growth medium (DMEM supplemented with 10% FBS and 0.1% penicillin/streptomycin). The following day, differentiation was induced with high-induction medium (growth medium, 20 nM insulin, 1 nM triiodothyronine, 1 μM Dexamethasone, 100 μM 3-isobutyl-1-methylxanthine, 1 μM Rosiglitazone and 125 nM Indomethacin). After 2 days, the medium was replaced with BA differentiation medium (growth medium, 20 nM insulin and 1 nM triiodothyronine). The following day, the respirometry Mito-Stress assay was performed according to manufacturer instructions, following 15 min of 7α,25-OHC (1 μM) treatment where indicated. The following substances were used: Norepinephrine (1 μM, Sigma-Aldrich), oligomycin (2 μM, Sigma-Aldrich), Carbonyl cyanide-4-(trifluoromethoxy)phenylhydrazone (FCCP, 1 μM, Tocris), Rotenone (0.5 μM, Tocris), Antimycin A (0.5 μM, Tocris), Hoechst staining (10 μg/ml, Sigma-Aldrich). The number of cells labeled with Hoechst staining was calculated with Cytation 5 Cell Imaging Multi-Mode Reader for normalization.

### Ex vivo respirometry

BAT samples were treated as indicated 15 min before oxygraph measurements (Oxygraph 2K; Oroboros Instru-ments). Samples were transferred to the oxygraph chamber containing 2 mL incubation medium (0.5 mM EGTA, 3 mM MgCl26H2O, 60 mM K-lactobionate, 20 mM taurine, 10 mM KH2PO4, 20 mM HEPES, 110 mM sucrose and 1 g/l BSA, pH 7.1). Respiration levels were recorded when reaching a steady state followed by addition of substrates (State 1: endogenous; state; 2: ADP; state3: succinate; state 4: oligomycin; uncoupled: FCCP). Respiration rates were normalized to total protein content.

### ROS measurement

Differentiated BAs were seeded on a black 96-well plate with clear bottom. The following day, the assay was performed according to manufacturer’s instruction in presence of 7α,25-OHC (1 μM, Avanti Biolipids), NIBR189 (10 μM, Tocris), Norepinephrine (1 μM, Sigma-Aldrich) or H_2_O_2_ (3%, Sigma-Aldrich). The fluorescence increase was measured at different time points at Ex/Em = 520/605 nm at bottom read mode with a EnSpire™ Multimode Plate Reader (Perkin Elmer).

### Immunohistochemistry

Standard hematoxylin/eosin staining was performed on 5 μm paraffin-embedded tissue sections after deparaffinization. For UCP1 staining, 5 μm paraffin-embedded tissue sections were blocked with 2.5% normal goat serum in PBST (Phosphate-buffer saline with 0.1% Tween 20) for 1 h at room temperature following deparaffinization. UCP1 primary antibody (1:50, Sigma-Aldrich) was applied overnight at 4 °C. The following day, secondary antibody against rabbit (SignalStain Boost IHC, Cell Signaling) was applied for 1 h at room temperature and developed with DAB kit (Vector Laboratories) according to the manufacturer’s instruction. For CH25H and perilipin staining, 5 μm paraffin-embedded tissue sections were incubated overnight at 4 °C with CH25H (1:50, Santa Cruz Biotechnology) or perilipin (undiluted, OriGene) primary antibody and stained with Tyramide SuperBoost Kit (Thermo Fisher) according to manufacturer’s instruction. Nucleus counterstaining was performed with NucRed 647 (Thermo Fisher).

### 7α,25-OHC measurements

7α,25OHC levels were analyzed using a validated HPLC-MS method^37^. Briefly, serum samples or tissue homogenates were placed in glass vials containing deuterated internal standards and dichloromethane, methanol and water in the presence of butylated hydroxytoluene (10 µg) and ethylenediaminetetraacetic acid (20 ng) to prevent oxidation. Following extraction, the lipid fraction was purified by solid phase extraction to remove cholesterol. The oxysterol fraction was analyzed by HPLC-MS using an LTQ-Orbitrap XL mass spectrometer (Thermo Fisher) coupled to an Accela HPLC system (Thermo Fisher). Chromatographic separation was performed using an Ascentis Express C-18 column (2.7 µm, 150 × 4.6 mm, Sigma), kept at 15 °C. Mobile phase was a gradient of methanol and water containing acetic acid. Calibration curves were prepared in the same conditions.

### Glucose and insulin measurement

Serum was collected after blood centrifugation (2000*g*, 10 min). 5 μl of serum were used to perform the assay according to the manufacturer’s instruction (Chrystal Chem). All the measurements were run in duplicate.

### Animals

8-week old wild-type male C57Bl/6J mice were purchased from Charles River Laboratories. EBI2 knockout (EBI2−/−) mice were kindly provided by Prof. Caroline Pot (Lausanne University Hospital CHUV, Lausanne). EBI2 floxed mice were provided by Dr. Stefano Casola (IFOM, Milan), and crossed with heterozygous LysM-Cre mice to obtain the mouse line M-EBI2−/−. Animals were given access to chow diet and water *ad libitum*, and maintained on a 12 h dark/light cycle. For all analysis, 8 weeks old male mice were used. All studies were approved by the Landesamt für Natur, Umwelt und Verbraucherschutz, Nordrhein-Westfalen, Germany (Animal protocol No. 84-02.04.2017.A311). All animals were housed at 23 °C ± 1 °C at the Haus für experimentelle Therapie, University Clininc Bonn, or at the Institute of Pharmacology and Toxicology, University Clininc Bonn, during experiments. For pharmacological studies, mice were injected i.p. with 7α,25-OHC or NIBR189 (5 mg/kg in 0.9% w/v NaCl with 10% DMSO for both compounds) or vehicle at the indicated temperature.

### In vivo energy expenditure measurement

EE (VO_2_) was measured with a TSE Phenomaster system at the indicated temperature. Animals were single caged for the whole measurement and were acclimatized for 24 h before recording.

### Infrared thermography

Infrared thermography is an established measure of brown adipose tissue activity^38^. Thermographic images were taken from newborn littermates at room temperature with an infrared camera (IC060, Trotec) and analyzed with IC-Report software 1.2 (Trotec).

### Statistical analysis and reproducibility

All data are presented as mean ± s.e.m. Statistical analysis was performed using a two-tailed Student’s *t* test (paired comparison for in vitro pharmacological treatments, unpaired comparison otherwise), two-way or one-way analysis of variance (ANOVA) with the post-hoc Tukey test for multiple comparisons. *P*-value smaller than 0.05 was considered significant. At least three independent cell cultures were used for all in vitro analysis. No technical replicates were used. The number of mice analyzed is stated in the respective figure legends. Statistical analysis was performed with Graph Pad Prism 6.

### Reporting summary

Further information on research design is available in the Nature Research Reporting Summary linked to this article.

## Supplementary information

Supplementary information

Description of Additional Supplementary Files

Supplementary Data 1

Reporting Summary

## Data Availability

The data that support the findings of this study are available from the corresponding author upon reasonable request. Uncropped Western blots are shown in Supplementary Fig. 7. Source data of the main figures are provided as Supplementary Data 1.
