# Supplementary material for: EBI2 is a negative modulator of brown adipose tissue energy expenditure in mice and human brown adipocytes
Source: Commun Biol. 2022 Mar 29;5:280. doi: 10.1038/s42003-022-03201-6 (PMC8964700; doi:10.1038/s42003-022-03201-6)
Supplement: Supplementary file 1 — Supplementary information [file 42003_2022_3201_MOESM1_ESM.pdf]

Supplementary information

EBI2 is a negative modulator of brown adipose tissue energy expenditure in mice and human brown adipocytes

Authors: Francesca Copperi[1,2], Inna Schleis[1], Martin Roumain[3], Giulio G. Muccioli[3], Stefano Casola[4], Martin Klingenspor[5,a,b], Alexander Pfeifer[1], Thorsten Gnad[1*]

Affiliation: [1]Institute of Pharmacology and Toxicology, University Hospital Bonn, University of Bonn, Bonn, 53127, Germany; [2]Research Training Group 1873, University of Bonn, Bonn, 53127, Germany; [3]Bioanalysis and Pharmacology of Bioactive Lipids Research Group, Louvain Drug Research Institute, UCLouvain, Université catholique de Louvain, 1200 Bruxelles, Belgium. [4]The FIRC Institute of Molecular Oncology (IFOM), Milan, 20139, Italy. [5]Molecular Nutritional Medicine, TUM School of Life Sciences, Technical University of Munich, 85354 Freising, Germany. [a]EKFZ - Else Kröner-Fresenius Center for Nutritional Medicine, Technical University of Munich, 85354 Freising, Germany. [b]ZIEL - Institute for Food & Health, Technical University of Munich, 85354 Freising, Germany.

* Corresponding author: tgnad@uni-bonn.de

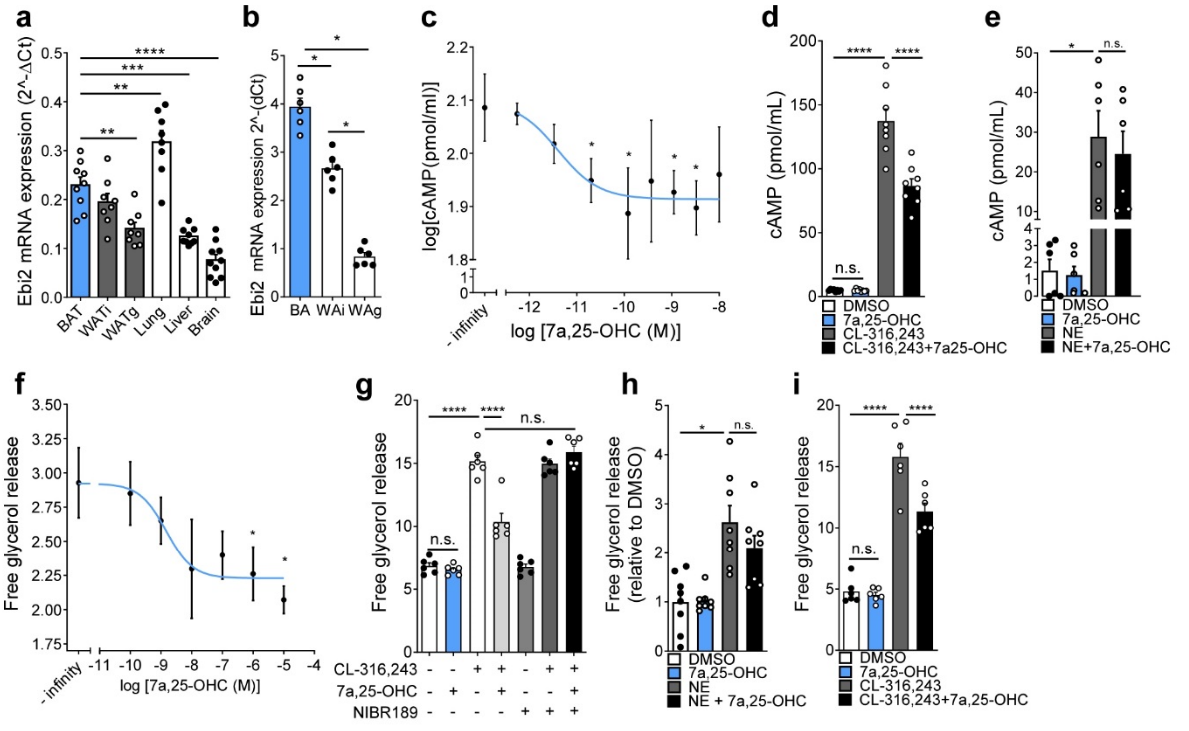

Supplementary Figure 1. 7α,25-OHC treatment of BA and WA

a EBI2 mRNA expression in ATs and other relevant organs (n=8-10). **b** EBI2 mRNA expression in adipocytes (n=6). **c** Dose dependent NE-induced intracellular cAMP decrease after 7α,25-OHC treatment of BA (n=7-14). **d** 7α,25-OHC (1μM) effect on CL-316,243-induced (1μM) intracellular cAMP in BA (n=8). **e** Intracellular cAMP levels in WA treated with NE (1μM) and 7α,25-OHC (1μM) (n=6). **f** Dose dependent NE-induced lipolysis decrease after 7α,25-OHC treatment of BA (n=3-9). **g** 7α,25-OHC (1μM) and NIBR189 (10μM) effect on CL-316,243-induced (1μM) lipolysis in BA (n=6). **h** Relative lipolysis of WA treated with NE (1μM) and 7α,25-OHC (1μM) (n=8). **i** 7α,25-OHC effect on CL-316,243-induced (1μM) lipolysis in hMADs (n=6). Mean ± s.e.m., one-way ANOVA with Tukey post-hoc test and two-way ANOVA, *p<0.05, **p<0.01, ***p<0.001, ****p<0.0001.

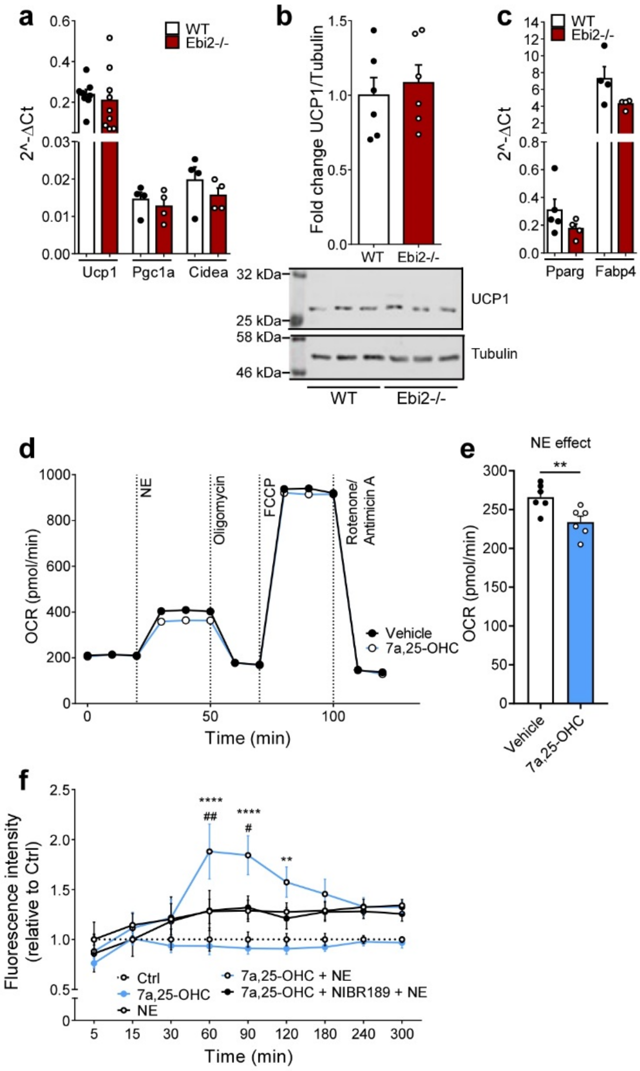

Supplementary Figure 2. Marker gene expression of EBI2-/- BA and ROS production after EBI2 activation
a Thermogenic genes mRNA expression in WT and EBI2-/- BA (n=4-10). **b** UCP1 immunoblot of WT and EBI2-/- BA (n=4). **c** Adipogenic genes mRNA expression in WT and EBI2-/- BA (n=4-5). **d**,**e** In vitro respirometry of human BA pre-treated with 7α,25-OHC (1µM) or vehicle (n=6). **f** Time course of ROS production by BA treated with NE (1µM), 7α,25-OHC (1µM), NIBR189 (10µM) and H2O2 (3%) (n=4). Mean ± s.e.m., student's t-test and two-way ANOVA, #p<0.05, ##/**p<0.01, ****p<0.00001.

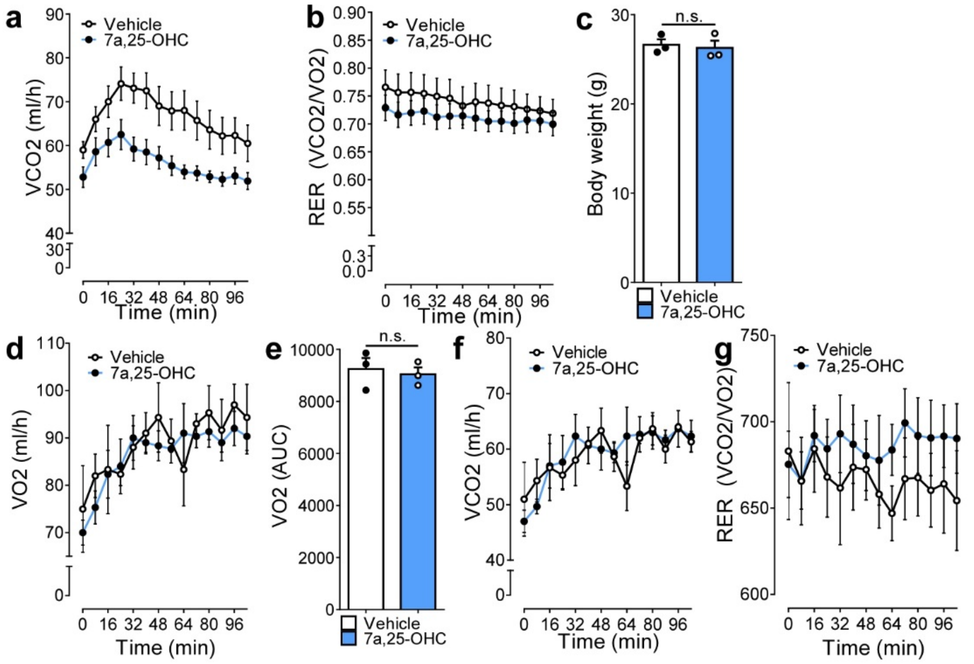

Supplementary Figure 3. Effects of EBI2 activation on whole body metabolism.
a-c CO2 production **a** RER **b** and body weight **c** of WT mice injected with 7α,25-OHC (5 mg/kg i.p.) (n=10 and n=3). **d-g** Energy expenditure (VO2) **d,e**, CO2 production **f**, and RER **g** after injection of 7α,25-OHC (5 mg/kg i.p.) in EBI2-/- mice (n=3). Mean ± s.e.m., student's t-test and two-way ANOVA.

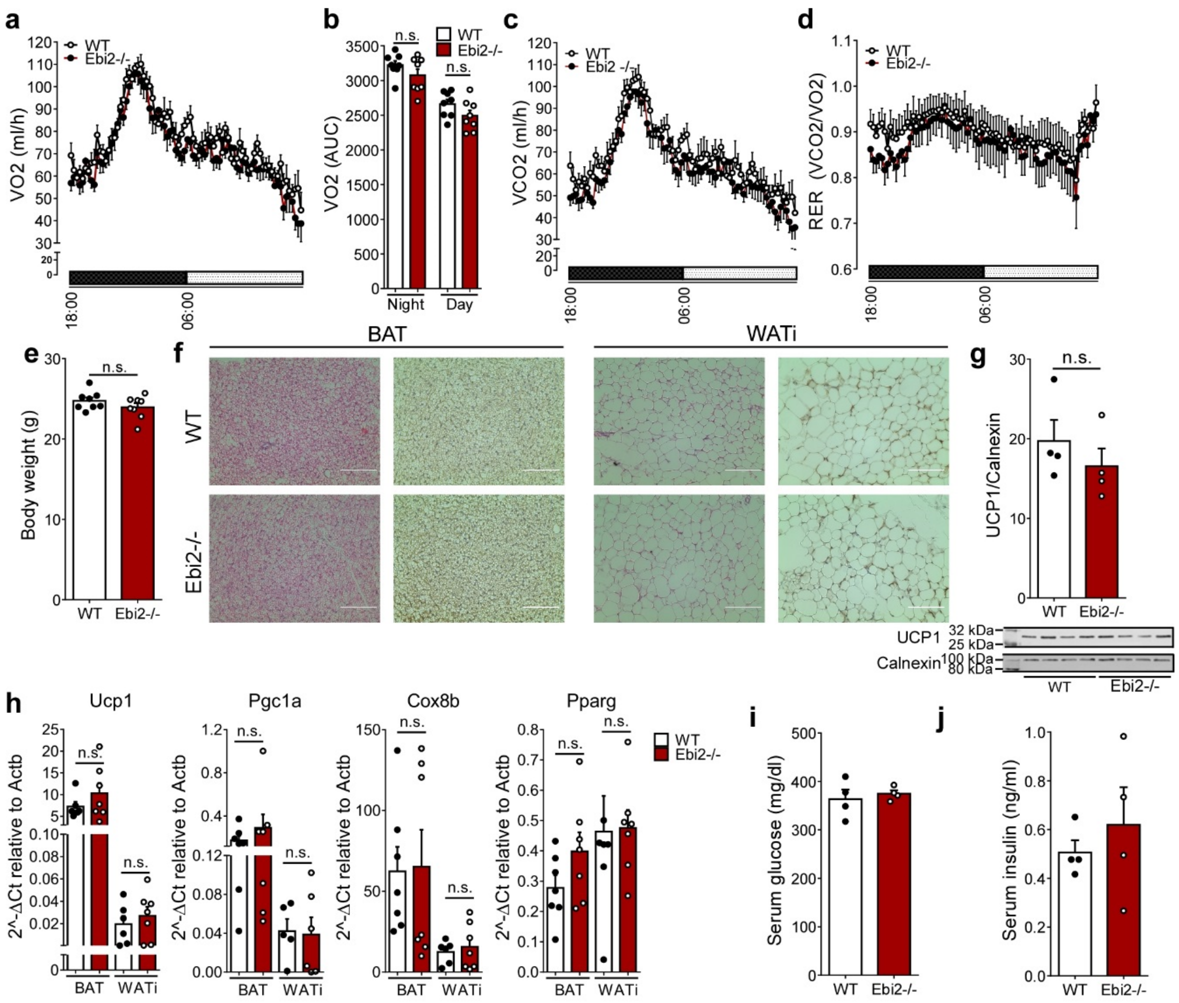

Supplementary Figure 4. Metabolism of EBI2-/- mice at 23°C
a-e Energy expenditure **a,b** CO2 production **c** RER **d** and body weight **e** of WT and EBI2-/- mice at 23°C (n=8). **f** HE and UCP1 staining of BAT and WATi of WT and EBI2-/- mice (scale bar 100 µm). **g** Representative UCP1 immunoblot and relative quantification in BAT of WT and EBI2-/- mice at 23°C (n=4). **h** Thermogenic and adipogenic genes mRNA expression in ATs of WT and EBI2-/- mice at 23°C (n=7). **i,j** Serum glucose **i** and insulin **j** levels of WT and EBI2-/- mice at 23°C (n=4). Mean ± s.e.m., two-way ANOVA and student's t-test.

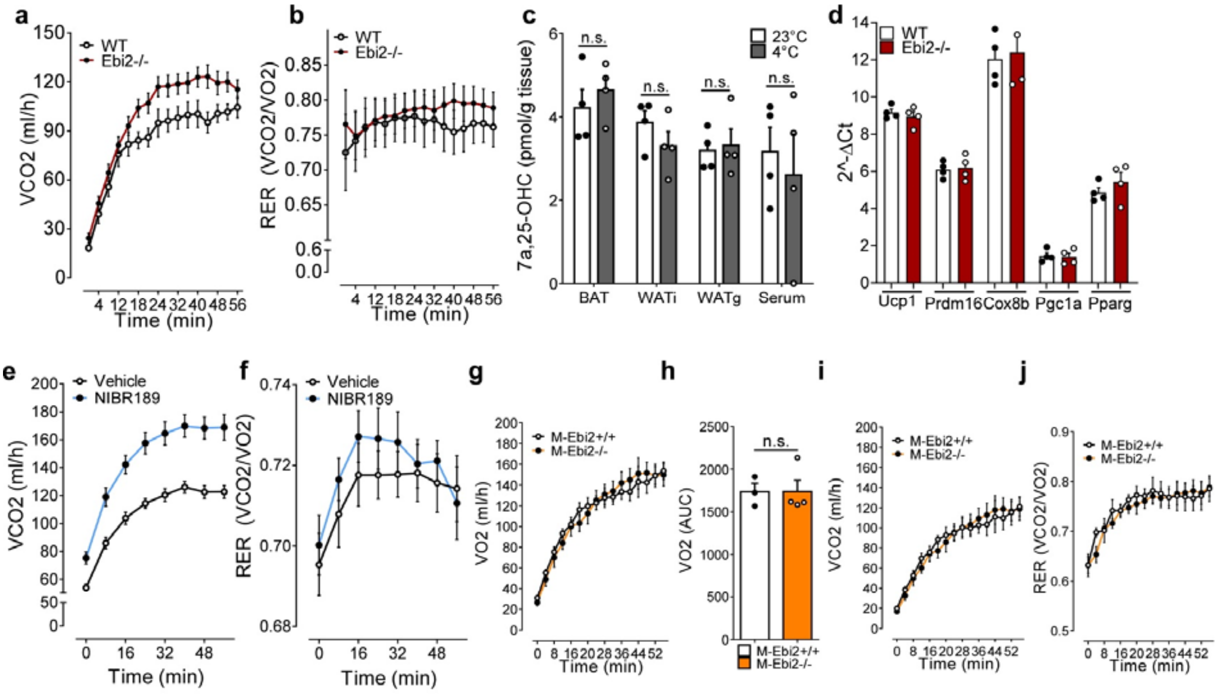

Supplementary Figure 5. Acute cold exposure of EBI2 mouse models

a,b CO2 production **a** and RER **b** of WT and EBI2-/- mice in acute cold exposure (4°C) (n=5-6). **c** 7α,25-OHC levels in ATs and serum before and after 1 h of cold exposure (4°C) (n=4). **d** Adipogenic and thermogenic genes expression upon 1h of cold exposure (4°C) of WT and EBI2-/- mice (n=4). **e,f** VCO2 **e** and RER **f** upon NIBR189 injection in acute cold exposure (4°C) (n=12). **g-j** O2 consumption **g,h** CO2 production **i** and RER **j** of M-EBI2+/+ and M-EBI2-/- mice during 1h cold exposure (4°C) (n=4). Mean ± s.e.m

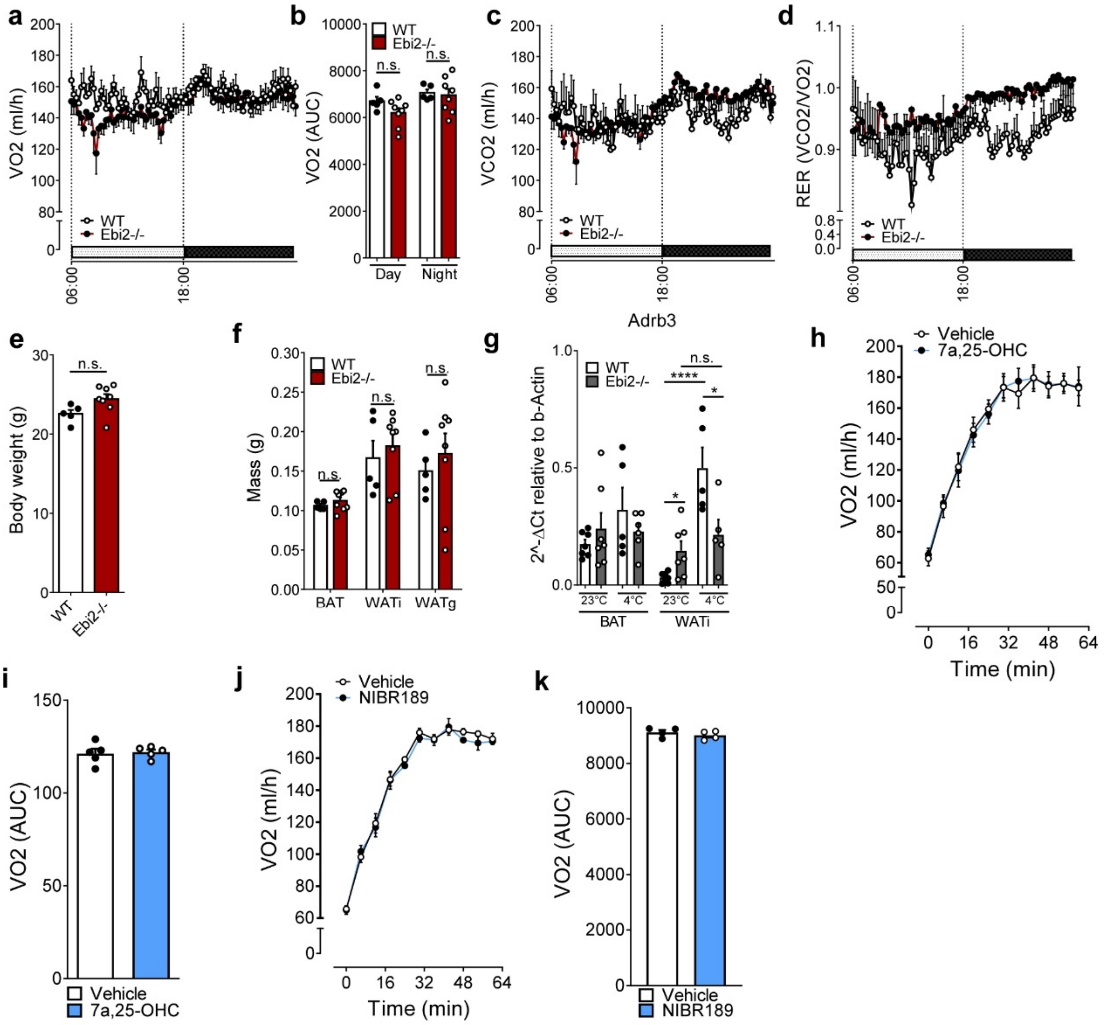

Supplementary Figure 6. Metabolism of EBI2-/- mice

a-b O2 consumption **c** CO2 production **d** RER **e** body weight **f** adipose tissues weight **g** and β3-AR expression in thermogenic fat depots of EBI2-/- or WT mice after prolonged cold exposure (1 week at 4°C) (n=5-8). **h-k** VO2 of EBI2-/- mice upon injection of 7α,25-OHC **h,i** or NIBR189 **j,k** after cold exposure (16°C) (n=4-5). Mean ± s.e.m., one-way and two-way ANOVA and student's t-test, *p<0.05, ****p<0.0001.

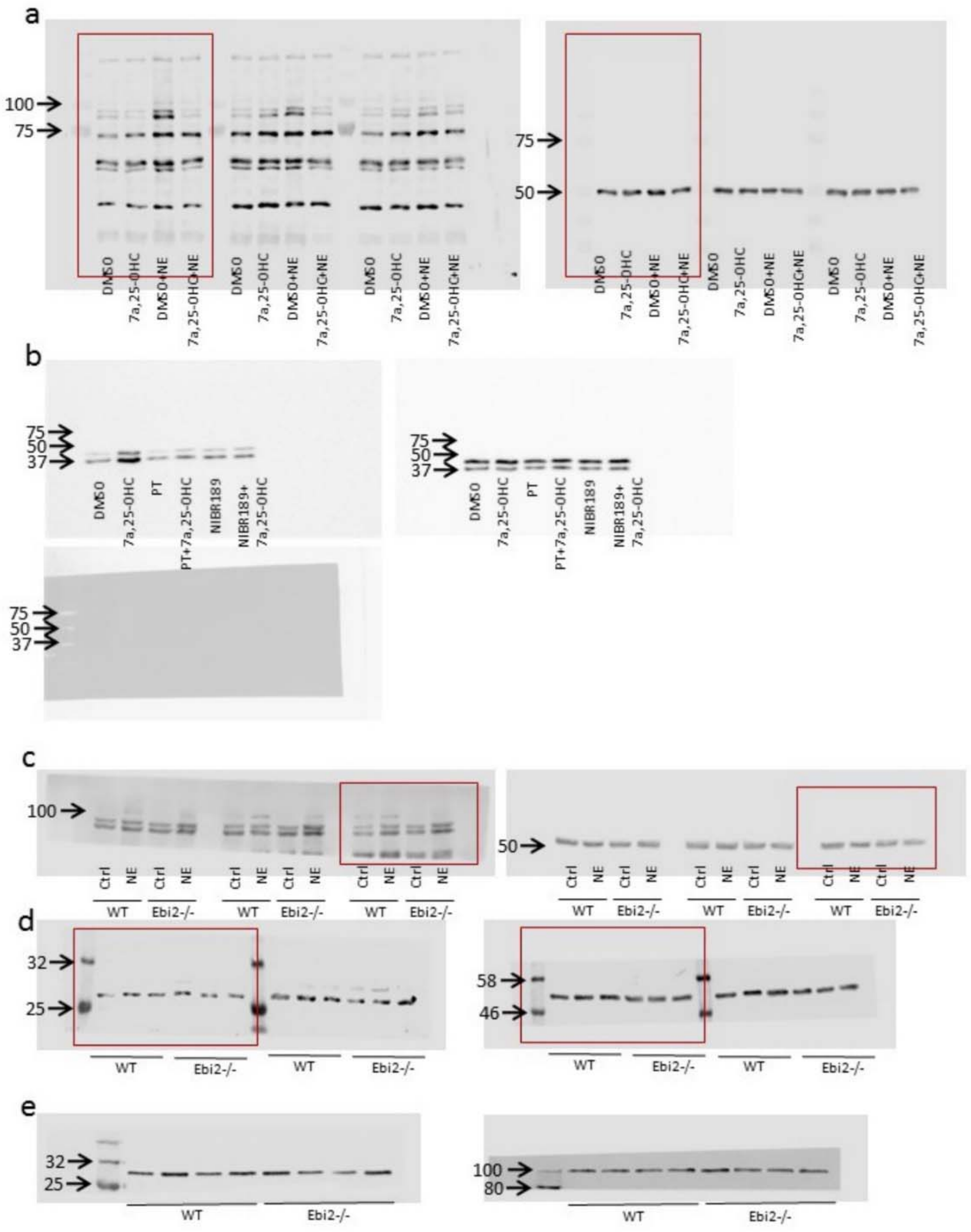

Supplementary Figure 7. **Uncropped Immunoblots. a** p-HSL (left) and tubulin (right), uncropped immunoblot shown in Figure 1f. **b** p-ERK1/2 (upper left), total-ERK1/2 (upper right) and ladder (bottom), uncropped immunoblot shown in Figure 1i. **c** p-HSL (left) and tubulin (right), uncropped immunoblot shown in Figure 2g. **d** UCP1 (left) and tubulin (right), uncropped immunoblot shown in Supplementary Figure 2b. **e** UCP1 (left) and calnexin (right), uncropped immunoblot shown in Supplementary Figure 4g. When other samples not presented in the figures were present on the same immunoblot, the part presented in the figures is framed in red.

Supplementary Table 1. Expression of Gi coupled GPCR in BAT

Gi GPCR	Abundance (2^-dCt)	s.e.m.
GPR183	0.2033	0.0754
CMKLR1	0.0547	0.0064
GPR81	0.0533	0.0068
Adra2b	0.0523	0.0043
C5AR	0.0383	0.0048
NyB4	0.0267	0.0094
GPR34	0.025	0.0069
SSTR4	0.0237	0.0041
S1PR1	0.0193	0.0027
SSTR1	0.0047	0.0009
CCR3	0.0043	0.0003
GPR18	0.0042	0.0002
Adora1	0.0034	0.0009
GABA B1	0.0025	0.0003
Adra2a	0.0025	0.001
CXCR2	0.0024	0.0004
CXCR6	0.0011	0.0001
CHRM4	0.0267	0.0094
AGTR2	0.0043	0.0003
