# Supplementary material for: EBI2 is a negative modulator of brown adipose tissue energy expenditure in mice and human brown adipocytes
Source: Commun Biol. 2022 Mar 29;5:280. doi: 10.1038/s42003-022-03201-6 (PMC8964700; doi:10.1038/s42003-022-03201-6)
Supplement: Supplementary file 2 — Description of Additional Supplementary Files [file 42003_2022_3201_MOESM2_ESM.pdf]

Description of Additional Supplementary Files

File name: Supplementary Data 1
Description: Source data used to generate the charts of the manuscript.
