# Supplementary material for: EBI2 is a negative modulator of brown adipose tissue energy expenditure in mice and human brown adipocytes
Source: Commun Biol. 2022 Mar 29;5:280. doi: 10.1038/s42003-022-03201-6 (PMC8964700; doi:10.1038/s42003-022-03201-6)
Supplement: Supplementary file 4 — Reporting Summary [file 42003_2022_3201_MOESM4_ESM.pdf]

nature portfolio

Corresponding author(s): Alexander Pfeifer

Last updated by author(s): Dec 8, 2021

Reporting Summary

Nature Portfolio wishes to improve the reproducibility of the work that we publish. This form provides structure for consistency and transparency in reporting. For further information on Nature Portfolio policies, see our Editorial Policies and the Editorial Policy Checklist.

Statistics

For all statistical analyses, confirm that the following items are present in the figure legend, table legend, main text, or Methods section.

n/a	Confirmed	
☐	☒	The exact sample size (n) for each experimental group/condition, given as a discrete number and unit of measurement
☒	☐	A statement on whether measurements were taken from distinct samples or whether the same sample was measured repeatedly
☐	☒	The statistical test(s) used AND whether they are one- or two-sided<br>*Only common tests should be described solely by name; describe more complex techniques in the Methods section.*
☒	☐	A description of all covariates tested
☐	☒	A description of any assumptions or corrections, such as tests of normality and adjustment for multiple comparisons
☐	☒	A full description of the statistical parameters including central tendency (e.g. means) or other basic estimates (e.g. regression coefficient) AND variation (e.g. standard deviation) or associated estimates of uncertainty (e.g. confidence intervals)
☒	☐	For null hypothesis testing, the test statistic (e.g. F, t, r) with confidence intervals, effect sizes, degrees of freedom and P value noted<br>*Give P values as exact values whenever suitable.*
☒	☐	For Bayesian analysis, information on the choice of priors and Markov chain Monte Carlo settings
☒	☐	For hierarchical and complex designs, identification of the appropriate level for tests and full reporting of outcomes
☒	☐	Estimates of effect sizes (e.g. Cohen's d, Pearson's r), indicating how they were calculated

Our web collection on statistics for biologists contains articles on many of the points above.

Software and code

Policy information about availability of computer code

Data collection	ABI StepOne Software 2.3, LAS 4000 Control Software, ZEN 2.3, TSE Phenomaster Software
Data analysis	ImageJ 1.48v, ABI StepOne Software 2.3, Microsoft Excel 2007, Graphpad Prism 6

For manuscripts utilizing custom algorithms or software that are central to the research but not yet described in published literature, software must be made available to editors and reviewers. We strongly encourage code deposition in a community repository (e.g. GitHub). See the Nature Portfolio guidelines for submitting code & software for further information.

Data

Policy information about availability of data

All manuscripts must include a data availability statement. This statement should provide the following information, where applicable:

- Accession codes, unique identifiers, or web links for publicly available datasets
- A description of any restrictions on data availability
- For clinical datasets or third party data, please ensure that the statement adheres to our policy

The data that support the findings of this study are available from the corresponding author upon reasonable request.

Field-specific reporting

Please select the one below that is the best fit for your research. If you are not sure, read the appropriate sections before making your selection.

☒ Life sciences ☐ Behavioural & social sciences ☐ Ecological, evolutionary & environmental sciences

For a reference copy of the document with all sections, see nature.com/documents/nr-reporting-summary-flat.pdf

Life sciences study design

All studies must disclose on these points even when the disclosure is negative.

Sample size	no sample size calculation was performed for in vitro analysis. Samples sizes for animal experiments were calculated using Power and Sample Size Calculator Version 3.0.43
Data exclusions	No data was excluded
Replication	Every experiment was repeated a minimum of three times to ensure reproducibility
Randomization	Due to the nature of the experiments, randomization of the samples was not applicable
Blinding	The investigators were not blinded to group allocation in this study

Reporting for specific materials, systems and methods

We require information from authors about some types of materials, experimental systems and methods used in many studies. Here, indicate whether each material, system or method listed is relevant to your study. If you are not sure if a list item applies to your research, read the appropriate section before selecting a response.

Materials & experimental systems

n/a	Involved in the study	
☐	☒	Antibodies
☐	☒	Eukaryotic cell lines
☒	☐	Palaeontology and archaeology
☐	☒	Animals and other organisms
☒	☐	Human research participants
☒	☐	Clinical data
☒	☐	Dual use research of concern

Methods

n/a	Involved in the study	
☒	☐	ChIP-seq
☒	☐	Flow cytometry
☒	☐	MRI-based neuroimaging

Antibodies

Antibodies used	p-HSL, Cell Signaling #45804<br>HSL, Cell Signaling, #4107<br>p-ERK Cell Signaling #4370<br>Erk, Cell Signaling #9102<br>UCP1 Sigma Aldrich<br>Tubulin ,Dianova DLN-09993<br>Calnexin, Novus Biologicals
Validation	Antibodies were tested by manufacturer

Eukaryotic cell lines

Policy information about cell lines

Cell line source(s)	Stromal vascular fraction cells were isolated from BAT and WAT of C57Bl/6 mice and differentiated to brown and white adipocytes as described in Haas et al.; 2009 and Gnad et al.; 2014; hMADS were provided by C. Dani (Nice, France).
Authentication	Cell lines were not authenticated
Mycoplasma contamination	Cell lines were not tested
Commonly misidentified lines<br>(See ICLAC register)	*Name any commonly misidentified cell lines used in the study and provide a rationale for their use.*

Animals and other organisms

Policy information about studies involving animals; ARRIVE guidelines recommended for reporting animal research

Laboratory animals	C57Bl6 mice (Charles River); EBI2 knockout mice were provided by Stefano Casola. LysMcre mice (Jackson #018956) Only male mice were analyzed.
Wild animals	*Provide details on animals observed in or captured in the field; report species, sex and age where possible. Describe how animals were caught and transported and what happened to captive animals after the study (if killed, explain why and describe method; if released, say where and when) OR state that the study did not involve wild animals.*
Field-collected samples	*For laboratory work with field-collected samples, describe all relevant parameters such as housing, maintenance, temperature, photoperiod and end-of-experiment protocol OR state that the study did not involve samples collected from the field.*
Ethics oversight	All studies were approved by the Landesamt für Natur, Umwelt und Verbraucherschutz, Nordrhein-Westfalen, Germany (Animal protocol No. 84-02.04.2017.A311).

Note that full information on the approval of the study protocol must also be provided in the manuscript.
